# The development of biologics to target SARS-CoV2: Treatment potential of antibodies in patient groups with poor immune response

**DOI:** 10.1016/j.crphar.2021.100064

**Published:** 2021-10-09

**Authors:** William Migo, Marko Boskovic, Robert Likic

**Affiliations:** a University of Zagreb School of Medicine, Croatia; b Clinical Hospital Centre Zagreb, Department of Internal Medicine, Division of Clinical Pharmacology and Therapeutics, Croatia

**Keywords:** COVID-19, SARS-CoV-2, Antibodies, Biologic drugs, Immunosuppression, Impaired immunity, Patients

## Abstract

Development of novel antibodies to combat the novel SARS-CoV-2 virus is ongoing. Importantly, particular subgroups are more prone to severe disease, namely patients with poor immune responses. This includes cancer patients with solid and haematological disease, solid organ transplant (SOT) patients and those with congenital or acquired immunodeficiency. Outcomes for patients with poor immune responses receiving antibody therapy for underlying disease and SARS-CoV-2 severe infection are undergoing investigation. The objective of this study was to perform a search on patients with poor immune responses with severe SARS-CoV-2 infection, to assess if antibody therapy is beneficial in such populations. We performed searches using PubMED and medrXiv up to May 2021 of patients with solid and hematologic malignancy, SOT patients and acquired or congenital immunodeficiency. The primary outcome was to assess if antibody therapy was included during SARS-CoV-2 infection and the clinical outcomes of such treatment in this population. Here we find that there is a repurposing of monoclonal antibodies to target cytokine release syndrome, along with the use of convalescent plasma (CP). Despite CP demonstrating promising results, we reiterate evidence that CP forces mutational escape and subsequent variant development. Repurposing of antibody therapies (such as Tocilizumab) proved effective, especially in SOT patients. This also potentially opens an avenue for the use of anti-SARS-CoV-2 spike protein neutralizing monoclonal antibodies; however, studies have yet to focus on patients with poor immune responses as a subpopulation.

## 1 Introduction

Severe Acute Respiratory Syndrome Coronavirus-2 (SARS-CoV-2) was declared a pandemic by the World Health Organisation (WHO) on March 11, 2020, (WHO, 2020). Its global spread has challenged the scientific community to repurpose existing monoclonal antibodies (mAb) and develop novel treatments. High risk patients with comorbidities and/or compromised immune systems are at greater risk for severe SARS-CoV-2 infection and represent an important subpopulation in need of appropriate care (Mehta et al., 2020). Generally, patients older than 60 years, with underlying medical comorbidities (obesity, cardiovascular disease, chronic kidney disease, diabetes, COPD) or immunocompromised states (solid and haematological malignancy, hematopoietic stem cell and solid organ transplant patients) were reported to have hospitalization rates 6 times higher compared to their healthy counterparts (45.4% vs 7.6%), (Stokes et al., 2020). In order to prevent higher mortality and address the needs of this at-risk sub-population, treatment regiments should broaden and improve.

Further, those with solid malignancy (especially lung cancer), solid organ transplant (SOT) or on chronic immunosuppressive disease modifying treatment had increased risk of severe outcomes such as secondary bacterial/fungal superinfection, higher ICU admissions, mechanical ventilation with intubation and overall mortality (Waghmare et al., 2016; Fung and Babik, 2020). Although this is true for the majority of immunocompromised states, we recognize there is heterogeneity, complexity and exceptions to this. For example, hematopoietic stem cell transplant (HSCT) patients demonstrate a similar severity score compared to their healthy counterparts (Albiges et al., 2020; Belsky et al., 2021). Whether this is due to their current immunosuppressive regimen has still yet to be fully elucidated and paints a more complex picture.

Patients with poor immune responses owing to underlying disease and/or immunosuppressive therapy are at increased risk for severe infection, often coupled with Cytokine Release Syndrome (CRS). CRS is characterised by fever, hyperferritinemia and a cytokine storm (namely, increased production of IL-6, TNF-a and monocyte chemoattractant molecules). Importantly, Huang et al. concluded that disease severity is correlated with hypercytokinaemia with characteristic increases in specific serum interleukin profiles in ICU patients (IL-2, IL-7, IL-10, G-CSF, MCP-1, IFN-y, TNF-a, ferritin and IL-6) compared to non-ICU patients (Wan et al., 2020; Huang et al., 2020; Coperchini et al., 2020). During these hyper-inflammatory states, SARS-CoV-2 patients were found to have an exhausted immune system with decreased monocyte and CD4+/CD8^+^ T cell count (lymphopenia) and pathogenic TH1 cells resulting in increased cytokine parameters. Overall, a lymphopenic and hypercytokinaemia profile definitively suggests a severe SARS-CoV-2 infection with poor prognosis and these inflammatory parameters are now used to guide treatment (Coperchini et al., 2020)

Interestingly, this association between SARS-CoV-2 infection and CRS has steered treatment towards immunosuppression via CRS specific mAbs benefiting patients by reducing proinflammatory reactivity and subsequently abating the storm. Additionally, a more preventative approach, especially significant in immunocompromised patients, might include Neutralizing mAbs (NMAbs) that target the SARS-CoV-2 receptor binding domain (RBD) and prevent severe disease developing.

Currently, several pharmaceutical companies are trialing repurposed mAbs that target the CRS and are under investigation in patients with poor immune responses (Table 1.1). There is also research demonstrating clinical efficacy with convalescent plasma (CP) administration in these patients. However, we must acknowledge the potential for mutational escape, especially recent data observing the emergence of multiple SARS-CoV-2 antibody escape variants in immunocompromised hosts undergoing CP treatment (Chen et al., 2021). Targeting both SARS-CoV-2 RBD with NMAb's/CP, while suppressing the CRS with repurposed mAbs, provides a robust multi-hit mechanism in preventing severe disease. Although the FDA recently granted emergency use authorization for CP in non-hospitalized patients with mild-to-moderate SARS-CoV-2 infection, it is still not known the extent to which mAb therapies will improve outcomes in at-risk patients. It is also recognized that the type of immunosuppressant therapy in these patients does not always correspond to severe disease and is increasingly complex. More research is needed in order to explore these intricacies and guide treatment plans in immunocompromised patients with SARS-CoV-2 infection.

**Table 1.1:** - Potential targets of the Cytokine Release Sydrome. See Fig. 1.1 to see each targetable step in the cytokine cascade. Adapted from Kim JS et al. (2021).

	Targeted Inhibition	Drugs/Intervention	Targeted Inhibition	Drug/Intervention
1	**IL-1**	**Anakinra, Canakinumab**	**Nonselective**	**Colchicine**
2	**IL-6**	**Tocilizumab, Sarilumab, Siltuximab**	**Nonselective**	**Mesenchymal stem cells**
3	**TNF-a**	**Etanercept**	**Nonselective**	**Plasma exchange**
4	**IFN-y**	**Emapalumab**	**Nonselective**	**IV Immunoglobulin**
5	**JAK**	**Baricitinib, Ruxolitinib**	**Nonselective**	**Covalescent plasma**
6	**Nonselective**	**Glucocorticoids**	**Nonselective**	**Radiation**

**Fig. 1.1:**
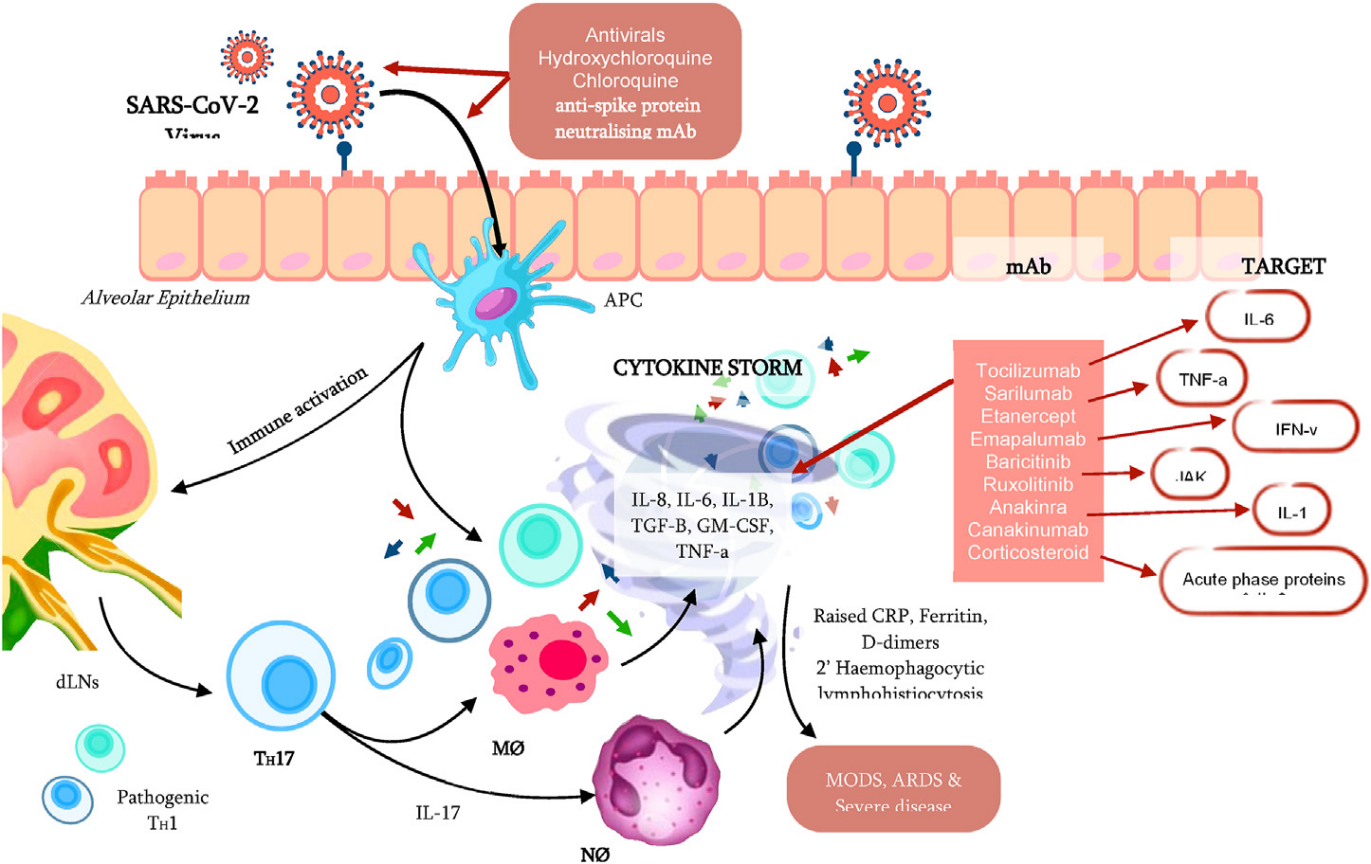
Diagram to illustrate targeted therapies against SARS-CoV-2 and the Cytokine Storm. Illustration adapted from Pelaia C et al. (2021). APC ​= ​Antigen Presenting Cell; dLNs ​= ​Draining Lymph Nodes; MOD ​= ​Multi-Organ Dysfunction; NØ ​= ​Neutrophil; MØ ​= ​Macrophage; TNF-a ​= ​Tumour Necrosis Factor-a; IFN-y ​= ​Interferon y.

The expansion of prophylactic therapy for SARS-CoV-2 in patients with poor immune responses is increasingly prudent. Poor vaccine immunogenicity and the growing threat of mutational escape globally is forcing the development of alternative targets in the form of immunotherapy as previously described. According to a recent review patients with haematological and solid cancers demonstrate reduced immunogenicity to SARS-CoV-2 vaccinations. This is a constant trend echoed amongst other vaccines, for example, acute lymphoblastic leukemia (ALL) patients had 10–27% immunogenicity after hepatitis B and meningococcal subunit vaccines (Yu et al., 2007). Further, annual influenza vaccinations for haematological cancer patients demonstrated 10–42% response (Goyal et al., 1998). Boyarsky et al. (2021) report 17% of patients developing anti-SARS-CoV-2 antibodies in response to an initial dose of BNT162b2 (Pfizer-BioNTech) or mRNA-1273 (Moderna). We acknowledge the limited data on this topic but clinical trials are underway, observing immunological response in immunocompromised patients (Table 1.2). Table 1.1 and Fig. 1.1 show potential targets of the CRS. Finally, it is agreeable that urgent discussion is required about the growing potential of novel mAb therapies, in order to develop strategies on how to use them effectively during clinical practice. Specifically, this relates to safety and production, availability and eventual distribution of mAbs. There is also a need to dissect the complex interactions of immunosuppressive drugs in simultaneously controlling underlying disease (for example chemotherapy for long-term rheumatic patients) and the SARS-CoV-2 CRS in an effort to improve understanding and clinical outcome in these patients. Clinicians are altering immunosuppressive drug regimens to understand this and find ways that do not compromise treatment of underlying conditions in these patients. Here we discuss the data and results surrounding targeted mAb treatment for severe SARS-CoV-2 patients with poor immune responses.

**Table 1.2:** Current mAbs under clinical investigation (*n ​= ​66*). Data collected from various sources: PubMed and antibodysociety.org. We have excluded clinical trials in phase 1 and preclinical trials in order to focus on therapies closer to market. Total number of monoclonal antibody trials (preclinical and clinical) *n ​= ​229*. *Last updated: 26/04/21*.

INN	Codename	Target	Status for COVID-19
Bamlanivimab	LY-CoV555, LY3819253	SARS-CoV-2	EUA granted in US on Nov 9, 2020
Etesevimab ​+ ​bamlanivimab	JS016, LYCoV016, LY3832479, CB6-LALA; combination with LY-CoV555/LY3819253	SARS-CoV-2	EUA granted in US on Feb 9, 2021
Casirivimab ​+ ​imdevimab	REGN-COV2 (REGN10933+REGN10987)	SARS-CoV-2	EUA granted in US on Nov 20, 2020
	TY027	SARS-CoV-2	Phase 3
	BRII-196	SARS-CoV-2	Phase 3 pending
	BRII-198	SARS-CoV-2	Phase 3 pending
Regdanvimab	CT-P59, Regkirona	SARS-CoV-2	Conditional use authorization granted in South Korea
	SCTA01, H014 (assumed)	SARS-CoV-2	Phase 2/3
	SAB-185	SARS-CoV-2	Phase 2/3
	ABP-300, MW33	SARS-CoV-2	Phase 2/3
Tixagevimab (AZD8895) ​+ ​Cilgavimab (AZD1061)	AZD7442 (AZD8895 ​+ ​AZD1061)	SARS-CoV-2	Phase 3
Sotrovimab	VIR-7831, GSK4182136	SARS-CoV-2	EUA requested in the EU and US
	DXP-593	SARS-CoV-2	Phase 2 pending
	XAV-19	SARS-CoV-2	Phase 2
	BI 767551, DZIF-10c	SARS-CoV-2	Phase 2/3 pending
	STI-2020 (IV administration), COVI-AMG; STI-2099, COVI-DROPS (intranasal)	SARS-CoV-2	Phase 2
	ADG20	SARS-CoV-2	Phase 2/3
Zansecimab	LY3127804	Ang-2	Phase 2
**Eftilagimod alpha**	IMP321	APCs	Phase 2
**Eculizumab**		C5	Phase 2
**Avdoralimab**	IPH5401	C5aR	Phase 2
**Abatacept**		CD80 and CD86	Phase 2
**Asunercept**	APG101	CD95 ligand	Phase 2
**Garadacimab**	CSL312	Factor XIIa	Phase 2
**Gimsilumab**		GM-CSF	Phase 2
**Otilimab**		GM-CSF	Phase 2
**Secukinumab**	AIN457	IL-17a	Phase 2
**Ixekizumab**		IL-17A	Phase 2
	UTTR1147A	IL-22R	Phase 2
	F-652	IL-22R	Phase 2
**Risankizumab**		IL-23 p19	Phase 2
**Astegolimab**	MSTT1041A, RG-6149, AMG-282	IL-33R	Phase 2
	MEDI3506	IL-33R	Phase 2
**Sirukumab**	CNTO 136	IL-6	Phase 2
	BMS-986253, HuMax-IL8, HuMax-Inflam/MDX018	IL-8	Phase 2
	CERC-002, AEVI-002, KHK-252067, SAR-252067	LIGHT	Phase 2
	ATYR1923	Neuropilin-2	Phase 2
**Monalizumab**	NN8765, IPH-2201, NNC141-0100	CD159a	Phase 2
**Camrelizumab**	PD-1 blocking antibody (?)	PD-1	Phase 2
**Nivolumab**		PD-1	Phase 2
**Pembrolizumab**		PD-1	Phase 2
**Crizanlizumab**		P-selectin	Phase 2
	APN01	SARS-CoV-2	Phase 2
	MAS825	TIM-3	Phase 2
**Infliximab**		TNF	Phase 2
	hzVSFv13	Vimentin?	Phase 2
**Atibuclimab**	IC14	CD14	Phase 2 pending
**Glenzocimab**	ACT017	Gp VI platelet	Phase 2 pending
	CMAB806	IL-6R	Phase 2 pending
**Vilobelimab**	IFX-1, BDB-001	C5a	Phase 2/3
**Pamrevlumab**	FG-3019	CCN2	Phase 2/3
**Plonmarlimab**	TJM2 (TJ003234)	GM-CSF	Phase 2/3
**Mavrilimumab**	KPL-301	GM-CSF receptor	Phase 2/3
**Clazakizumab**		IL-6	Phase 2/3
**Olokizumab**		IL-6	Phase 2/3
	RPH-104	Interleukin-1	Phase 2/3
	EB05, NI-0101	Toll-like receptor 4	Phase 2/3
**Bevacizumab**		VEGF	Phase 2/3
**Meplazumab (may not be offical INN)**		CD147	Phase 2/3 pending
**Ravulizumab-cwvz**		C5	Phase 3
	CPI-006, CPX-006	CD73	Phase 3
**Lenzilumab**		GM-CSF	Phase 3
**Siltuximab**		IL-6	Phase 3
**Tocilizumab**		IL-6R	Phase 3
	AR-301	S. aureus alpha-toxin	Phase 3

## 2 Search methods/methodology

**Fig. 1.2:**
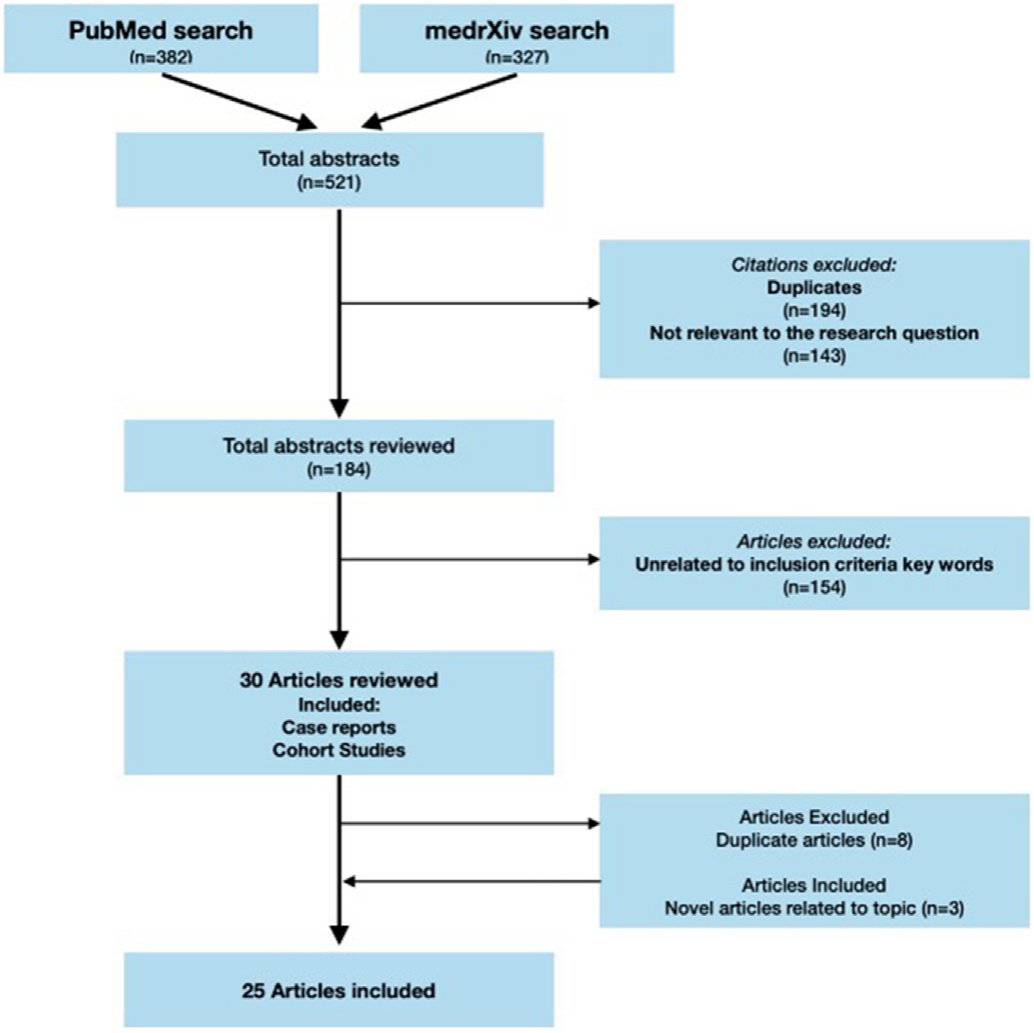
PRISMA Diagram, illustrating search methods with inclusion and exclusion process.

In order to attain a large volume of literature on this topic, we utilised PubMed and medrXiv search databases for publications on the use of antibody therapies in SARS-CoV-2 patients with poor immune responses from January 1st, 2019 to May 1st, 2021, see PRISMA digram (Fig. 1.2) which displays our search methods with exclusion and inclusion criteria. Patient's with poor immune responses included (but not limited to): oncological patients (including haematological patients), transplant patients and genetic or congenital immunodeficiency syndromes/conditions. Key words used in each search were: “COVID-19”, “SARS-CoV-2”, “convalescent plasma”, “neutralizing antibodies”, “antibodies”, “immune deficiency”, “monoclonal antibodies”, “cancer”, “hypogammaglobulinaemia”, “immunocompromised”, “transplant”, “kidney transplant”, “heart transplant”. Importantly, we also utilised reference lists from other review/primary research articles in order to attain additional relevant information previously not captured from our initial search. We primarily focus on clinical outcome, side effect profile and the efficacy of drug administration.

### 2.1 Production of mAbs

**Fig. 1.3:**
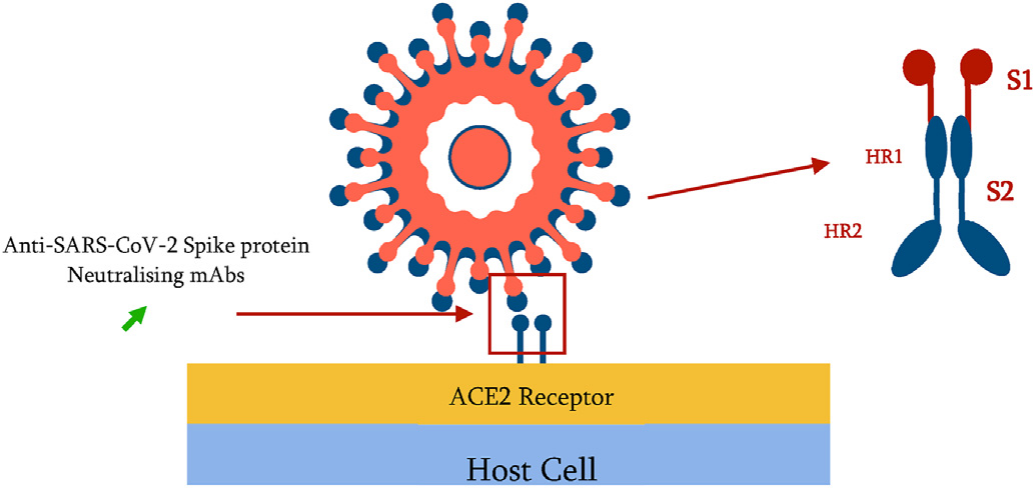
SARS-CoV-2 Receptor structural domains, S1 and S2 domains. SARS-CoV22 binding to ACE2 Receptor with interaction of Neutralizing mAbs (NMAbs). Diagram adapted from Huang Y et al. (2020).

The process of creating a novel mAb is complex, composing of animal and human testing. Firstly the target is identified for the antibody. NMAbs inhibit SARS-CoV-2 through inhibiting the viral spike protein (S-protein, specifically S1 subunit, Fig. 1.3) and subsequent viral entry ( Hansen et al., 2020; Lan et al., 2020; Huang et al., 2020).

After identifying a target, validation is confirmed through animal models using Bacterial Artificial Chromosomes (Bac) (Shizuya and Kouros-Mehr, 2001). This is where Bac is inserted into the genome in order to encode and produce humanized variable regions responsible for antibody production. The newly altered genome, or BacVec is then delivered into mice embryonic stem cells (ESCs), which replaces a portion of the mouse's original genome (Capecchi, 1989).

ESCs are filtered and selected for desired genetic modifications. These are inserted into murine embryos at an early stage of development. This results in subsequent murine progeny having the ability to create novel antibodies. Next, the target protein can be introduced.

One introduced, the target protein is identified and the new genome codes for the production of new antibodies. These are harvested and filtered via flow cytometry. Once viable antibodies are discovered, they are tested firstly in preclinical trials (safety and efficiency) and finally onto human trials.

### 2.2 Antibody therapy in patients with poor immune responses

Table 1.3 shows current clinical trials investigating the effect of mAb therapy in patients with poor immune responses with SARS-CoV-2 infection, with mainly cancer patients being investigated. Patient's with poor immune responses, for the purpose of this investigation includes (but not limited to): Solid and haematological cancers; Congenital & Acquired Immunodeficiencies; SOT patients.

**Table 1.3:** Current clinical trials investigating the effect on monoclonal antibody treatment of severe SARS-CoV-2 infection in patients with poor immune responses. Data collected from clinicaltrials.gov.uk.

Patient Population	Clinical Trial Title	NCT	Phase
Cancer	COVID-19 Prevention and Treatment in Cancer; a Sequential Multiple Assignment Randomised Trial; (C-SMART)	NCT04534725	3
	Prospective Study in Patients With Advanced or Metastatic Cancer and SARS-CoV-2 Infection (IMMUNONCOVID)	NCT04333914	2
	Tocilizumab for Patients With Cancer and COVID-19 Disease	NCT04370834	2
	COVID-19 Prevention and Treatment in Cancer; a Sequential Multiple Assignment Randomised Trial	NCT04534725	3
SOT	Multicentric Study of Coronavirus Disease (2019) (COVID-2019) in Solid Organ Transplant Recipients (COVIDSOT)	NCT04319172	N/A

Importantly, we recognize that immunosuppressive therapies have differing mechanisms of action on the immune system. A recent meta-analysis found no evidence of improvement of severe SARS-CoV-2 disease under treatment with TNF-blockers, IL-1 blockers or IL-6 blockers (Haberman et al., 2020; Russell et al., 2020). This perhaps suggests the requirement for a specific deficiency or abnormality in humoral immunity that then predisposes to severe disease as opposed to complete immunosuppression. This creates a more intricate roadmap and should be interrogated further. From our review of existing literature and ongoing clinical trials to date, we can see that there may be some benefit in modifying immunosuppressive regimens in order to control SARS-CoV-2 severe disease.

### 2.3 Solid & haematological cancers

As previously mentioned, data demonstrates patients with solid and haematological cancers are more prone to severe presentations, outcomes and higher mortality rates in comparison to general population (Kuderer et al., 2020; Lee et al., 2020; Tian et al., 2020, Mehta et al., 2020). Further, recent cancer treatment did not seem to significantly increase the risk of death (Vijenthira et al., 2020). One important clinical question is how to manage patients who need anticancer therapy during SARS-CoV-2 infection.

Rnjak et al. (Rnjak et al., 2021) demonstrate an improvement of clinical outcome in a patient with nasopharyngeal diffuse large B cell lymphoma with recurrent SARS-CoV-2 infection. Following administration of CP, the patient's symptoms resolved (afebrility, CRP and CXR lung infiltration regression). Importantly, when SARS-CoV-2 infection returned, re-administration of CP again resulted in regression of disease parameters (ferritin, IL-6 etc). The coinciding reduction in symptoms upon CP administration suggests a potential use for SARS-CoV-2-specific antibodies as a form of long-term chronic therapy in these patients. This treatment, in effect, enables the immune system time for immune reconstitution.

Currently, there are few clinical trials investigating the effect of mAbs on SARS-CoV-2 infection in solid and haematological cancer patients. The C-SMART trial (NCT04534725) is evaluating the effect of Selinexor and Lenzilumab (vs Placebo) on the treatment of SARS-CoV-2 infection in cancer patient's with moderate and severe SARS-CoV-2 infection respectively. Selinexor inhibits nuclear export while Lenzilumab is a mAb that targets CSF2/GM-CSF. This is a multi-centre trial with four main arms, expecting to provide insight into the best practice for prevention and treatment of SARS-CoV-2 in cancer patients as emerging standard of care measures are not always suitable to this especially vulnerable population.

Another multicenter randomised control study (NCT04333914) is investigating the efficacy of three mAbs: GNS561 (an autophagy inhibitor), Monalizumab (anti-NKG2A) and Avdoralimab (anti-C5aR), compared to the standard of care in patients with advanced or metastatic cancer and SARS-CoV-2 infection – results are expected toward the end of 2021. Finally, a recent phase two trial (NCT04370834) assessed the effect of Tocilizumab in reducing inflammation associated with CRS seen in severe SARS-COV-2 infection in cancer patients. However, recent randomised data no longer supported its continuation.

Overall, despite the positive results in many case studies/case-reports, there is still a lack of data surrounding cancer patient use of monoclonal antibodies to treat SARS-CoV-2 proinflammatory CRS. Specifically, large randomised control trials are needed in order to draw more robust and reliable conclusions.

### 2.4 Congenital & Acquired Immunodeficiencies

We have established patients lacking humoral response develop prolonged and a more severe course of infection. Since the onset of the pandemic, there is some evidence of the beneficial effects of mAbs and CP for patients with poor immune responses. Delgado-Fernandez et al. (Delgado-Fernández et al., 2021) report highly effective response rate from CP administration, demonstrating reduced relapse rates and chronicity (Kemp et al., 2021). Further studies indicate that immunocompromised patients at early stage of SARS-CoV-2 infection and no detectable anti-SARS-CoV-2 IgG are potential candidates for treatment with CP. Roman Rodionov et al. report well-tolerated infusions with 8 of 14 patients, showing clinical improvement on day 5. Subsequently, 86% of patients were discharged from hospital. Interestingly, they also found a correlation between the serum level of anti-SARS-CoV-2 IgG following the last transfusion and the degree of clinical improvement on day 5 – potentially acting as a guide for treatment with CP (Rodionov et al., 2021).

A recent study published by Leelayuwatanakul et al. (2021) observed the effect of Tocilizumab, IVIG and haemoperfusion in a favipiravir-based regimen for two patients with hypoxaemic respiratory failure requiring mechanical ventilation. Both patients had history of immunodeficiency (HIV and Multiple Myeloma). Here, the patient with history of HIV infection showed dramatic improvement upon administration of Tocilizumab. On the other hand, the patient with multiple myeloma showed a reduced response to treatment but eventually was extubated and discharged 45 days later upon additional treatment. Currently it is advised, in the case of extremely high IL-6 levels, to repeat dosing. However, authors here believe that the risk of repeated dose might outweigh the benefits. However, there is a lack of control trials on the use of IL-6 inhibitors in immunocompromised hosts with SARS-CoV-2 infection. Therefore, the risk-benefit profile of using tocilizumab should be carefully considered on an individual basis, until randomised control trials become more numerous.

### 2.5 Solid organ transplant patients

Due to transplant patient's increased frailty and immunosuppression in comparison to the general population, they are considered a high risk population (Guillen et al., 2020). The use of monoclonal antibody therapy in solid organ transplant (SOT) patient's during SARS-CoV-2 infection has been reported in several case studies - however larger randomised control trials have yet to be completed/conducted - see Table 1.3 for current mAbs in patients with poor immune responses in the treatment of SARS-CoV-2.

CP administration has demonstrated beneficial effects in SOT patients with improved therapeutic response to severe SARS-CoV-2 infection. Naeem et al. (2020) report improved clinical outcome in three SOT patients following CP administration[43]. Before receiving CP, patients developed severe SARS-CoV-2 infection (some requiring intubation). However, upon administration of CP all patients recovered without significant sequelae/side effects relating to CP. Prednisone was maintained while other immunosuppressive drugs were discontinued on admission as prednisone's anti-inflammatory effects act to minimise the alveoli infiltration, reducing the risk of acute respiratory distress syndrome (ARDS) and subsequent potential CRS (Naeem et al., 2021).

MAbs have also demonstrated improved clinical outcome in SOT patients. A recent case study observed effect of tocilizumab in a series of kidney transplant patients with severe respiratory SARS-CoV-2 infection. Despite 4 of 6 patients eventually succumbing to their underlying condition, C-reactive protein was reduced in all patients without significant modification in LDH or D-Dimer. IL-6 was reduced in 4/6 patients. Furthermore, in the 2 surviving patients, a significant increase in lymphocyte count was observed, supporting the notion that restoring lymphocyte activity is critical for obtaining a favourable outcome (Mella et al., 2020). Another case study conducted by Faguer et al. (Faguer et al., 2020) observed the effect of tocilizumab in a kidney transplant patient who developed haemophagocytic syndrome following SARS-CoV-2 infection. Upon multi-organ failure, administration of tocilizumab resulted in dramatic improvement and rapid reversal of respiratory, haemodynamic and liver conditions and correction of ferritin levels (Faguer et al., 2020). Another recent case study showed complete recovery from SARS-CoV-2 illness in a heart transplant patient following mAb administration. After rapid respiratory deterioration with increased inflammatory markers (CRP/IL-6), the patient received 25 ​mg IV clazakizumab, an IL-6 inhibitor now in phase 2/3 clinical trials (NTC04348500 and NTC04343989). The patient subsequently improved and was later discharged (Vaidya et al., 2020). This was echoed in another case report describing recovery of a heart transplant patient receiving tocilizumab (Mattioli et al., 2020). Belsky et al. (2021) conducted a recent meta-analysis of patients with poor immune responses exploring current therapeutic management. Accordingly, IL-6 blockage was reported the most amongst subpopulations and especially in SOT populations (n ​= ​120). They also noted a common therapeutic intervention whereby baseline immunosuppression was either modified or delayed.

Currently, a multicentric clinical trial is aiming to better understand the incidence, risk factors, etiology, clinical manifestations and outcome of SARS-CoV-2 infection in SOT patients (Multicentric Study of Coronavirus Disease 2019) (COVID-2019) in Solid Organ Transplant Recipients - Full Text View - ClinicalTrials.gov, 2021). These results will give researchers and clinicians a much needed insight into the potential future therapeutic strategies, including the need of antiviral treatment, how to adjust immunosuppressive therapy and indeed whether mAb therapies can be of use.

### 2.6 Safety

Repurposed mAbs operate through the alteration of the cytokine chain, such as tocilizumab, an IL-6 inhibitor. The complexity of the cytokine chain provides opportunities for the discovery of novel treatments (Fig. 1.1). Conversely, changes in the inner workings of the cytokine chain are difficult to predict and can yield severe and even fatal consequences. The adverse reactions of repurposed mAbs and the associated safety concerns can be divided into two sub-categories based on pharmacokinetics and severity.

Firstly, direct reactions to the administered proteins can cause anaphylactic reactions or cytokine storms (Estruch, 2011), both of which have potentially fatal consequences. This was the case in the trial of TGN1412 (a CD28 agonist antibody), 2006, where a life-threatening cytokine storm, not predicted by pre-clinical safety testing, occurred in all six healthy volunteers (Eastwood et al., 2010).

Indirect adverse events are more frequent and derived from the action of the antibody (opportunistic infections, common infections or development of autoimmune phenomena (Estruch, 2011).

As NMAb's have a specific target in the RBD and not in the cytokine chain, the reports from large SARS-COV-2 clinical trials have included primarily mild adverse events. In the specific case of bamlanivimab (a NMAb that targets SARS-CoV-2 RBD), the most frequent adverse events reported to the FDA have been nausea, dizziness, headache, pruritus, non-severe immediate hypersensitivity, diarrhea, and vomiting (FDA, 2021). Bamlanivimab has since been revoked by the FDA for use alone in mild-moderate SARS-CoV-2 infection, due to increasingly resistant SARS-CoV-2 strains (FDA, 2021). Moreover, few complications were reported in the REGN-COV2 trial where 2 of 93 patients (2%) in the placebo group and in 2 of 176 patients (1%) in the combined REGN-COV2 dose groups reported adverse events that were deemed serious or of special interest (Weinreich et al., 2021). REGEN-GOV (Previously known as REGN-COV2 - a combination of casirivimab and imdevimab mAbs) was approved for the use in mild to moderate COVID-19 in people aged twelve years of age and older weighing at least 40 ​kg (88 lb) with positive results of direct SARS-CoV-2 viral testing, and who are at high risk for progression to severe SARS-CoV-2 infection, including hospitalization or death. Adverse events included Infusion- and injection-related reactions and anaphylaxis (FDA, 2021).

A safety concern related to the preventing the spread of SARS-CoV-2 is the emergence of viral escape mutations. Viral escape is when under the pressure of medication, the virus mutates in order to evade neutralization. As S-protein variants that resist neutralization are now present at low frequencies in circulating SARS-CoV-2 populations, monoclonal antibody combinations as opposed to polyclonal CP are favored to mitigate viral escape (Weisblum et al., 2020). The combination NMAbs such as BRII-196/BRII-198 and REGN-COV2 are stated to prevent viral escape by binding to two distinct neutralizing epitopes (Hansen et al., 2020). In this instance, in order for viral escape to take place, two simultaneous viral mutations at two separate distinct genetic sites would need to take place.

## 3 Conclusion

Here we review the latest data on monoclonal antibody therapy in patients with poor immune responses following severe SARS-CoV-2 infection. It is evident that the number of studies conducted is limited. Results from case reports and cohort studies in this review tell us how treatment regimens of underlying conditions are being altered on an individual basis in order to prevent the otherwise deleterious effects of complete treatment cessation.

Tocilizumab appears to be leading SARS-CoV-2 mAb therapy, with ability to control CRS and reduce proinflammatory markers with subsequent resolution of severe disease. Despite inadequate data, CP also appears to prevent SARS-CoV-2 CRS. However, it is important to remain cautious due to increased mutational escape rates resulting from increased selective pressures (Weisblum et al., 2020; Rnjak et al., 2021).

Currently, it seems that clinical trials are focused on the effect of SARS-CoV-2 infection in immunocompromised patients clinically (i.e severity, response to current therapies and clinical outcome) and their ability to mount an immune response to current vaccinations. Outcomes of such patients are improving owing to biological therapies that target immune response against malignant disease, as well as supportive care and cellular therapies seen in SOT patients. These same treatments are being used in patients with severe SARS-CoV-2 infection in order to control the CRS clinical picture. Importantly, alterations in immunosuppressive therapies could negatively impact primary disease outcomes in these patients.

Overall, we recognize that the field surrounding this topic is rapidly evolving. With our discussion and conclusions based on case reports and cohort studies, we acknowledge the potential for accompanying biases. However, this will change as clinical data accumulates and more in-depth and large clinical trials are conducted within this subpopulation. Clinical trials should remain vigilant and continue to investigate the effect of MAbs for the treatment of severe SARS-CoV-2 infection in patients with poor immune responses.

## Funding

No funding support was provided for this study.

## CRediT authorship contribution statement

**William Migo:** conducted online search studies, Formal analysis, Writing – original draft. **Marko Boskovic:** Writing – original draft. **Robert Likic:** devised and suggested the topic, advised on all parts of the manuscript.

## Declaration of competing interest

The authors declare that they have no known competing financial interests or personal relationships that could have appeared to influence the work reported in this paper.
